# Roles of ROS in physiological, microbial and metabolomic alterations of fresh–cut sugarcane under red and blue light irradiation

**DOI:** 10.1016/j.fochx.2025.102344

**Published:** 2025-03-04

**Authors:** Lu Wang, Zhengrong Lin, Cheng Peng, Hua Zhang, Lulu Zhang, Shoujing Zheng, Jiebo Chen

**Affiliations:** a National Engineering Research Center of Sugarcane, College of Food Science, Fujian Agriculture and Forestry University, Fuzhou 350002, China; b Jinshan College of Fujian Agriculture and Forestry University, Fuzhou 350002, China

**Keywords:** Fresh-cut sugarcane, Red and blue light, Reactive oxygen species, Metabolomics

## Abstract

Effects of red and blue light treatment on physiological quality, microbial loads, redox status and metabolomics profiles of fresh-cut sugarcane in vacuum and plastic packages were investigated during 15 d storage. The results showed that light synergistic vacuum treatment delayed the decrease of pH and the increase of respiration rate and microbial loads, enhanced antioxidant capacities and related enzymes activities. Light treatment was beneficial to ^1^O_2_ generation, but had opposite effects on O_2_^−^, H_2_O_2_ and malondialdehyde. O^2−^ and H_2_O_2_ was negatively associated with CAT, sucrose, fructose, glucose, 2-oxoglutaramate, liquiritigenin and dihydromyricetin, positively with PPO and malondialdehyde. Only phenylacetaldehyde exhibited a negative correlation with ^1^O_2_. The biosynthesis of sugars, amino acids and flavonoids were the principal metabolite pathways corresponding to oxidative stress in fresh-cut sugarcane. It could be concluded that the concentration of ROS, especially O^2−^ and H_2_O_2_, should be appropriate to kill bacteria and retain the quality of fresh-cut sugarcane.

## Highlights

• LED synergistic vacuum delayed quality deterioration of fresh-cut sugarcane.
• LED was beneficial to ^1^O_2_ generation, but had opposite effects on O_2_^−^ and H_2_O_2_.
• Only phenylacetaldehyde exhibited a negative correlation with ^1^O_2_.
• Sugars, amino acids and flavonoids biosynthesis was activated under oxidative stress.
• ROS should be appropriate to kill bacteria and retain fresh-cut sugarcane quality.

## 1 Introduction

Sugarcane (*Saccharum officinarum* L*.*) is one of the most important sugar crops in tropical countries. In addition to sugar production, many sugarcanes are also consumed as a kind of edible fruit due to its nutritional value and sweet flavor (Wang, Deng, et al., 2020). Owing to the considerable length of sugarcane stalks and the difficulty in peeling them, the demand for fresh-cut sugarcane has increased in recent years. However, extending the shelf life of fresh-cut sugarcane are still great challenges because of the mechanical damage and microbial spoilage. Recently, many innovative attempts have been conducted to inhibit the quality deterioration of sugarcane including chemical additives and package materials. Singh et al. (2008) used glutaraldehyde and benzalkonium chloride to reduce postharvest sucrose loss and microbial infestation in sugarcane. Homaida et al. (2017) reduced browning and microbial growth by immersing fresh-cut sugarcane in ethanol. However, the potential chemical residues might not be preferred by consumers. Luo et al. (2014) found the beneficial effects of nano-CaCO_3_-LDPE packaging on sustaining the quality of fresh-cut sugarcane. Nevertheless, the cost and environmental protection property of these package should not be ignored. Therefore, it is urgent to find a safe, environmentally friendly and cost-effective preservation method to improve the storage quality of fresh-cut sugarcane.

Light-emitting diode (LED) has been recognized as an economical and energy efficient technology for improving the quality and prolonging shelf life of fresh-cut fruits and vegetables, such as pineapple (Bhavya et al., 2021) and spinach (Toledo et al., 2003). Lights with different wavelength might play deferent roles in maintaining quality of food. Early studies have shown the potential application of blue (400–500 nm) LED in food safety. Ghate et al. (2013) compared the inactivation effects of LED in the visible region (461, 521 and 642 nm) on foodborne pathogenic bacteria and found that LED with the wavelength of 461 nm was the most effective. Red (600–700 nm) light exhibited remarkably efficient in retaining bioactive compounds and antioxidant capacity to defense senescence (Nassarawa et al., 2022). The sensory appearance and chlorophyll of broccoli were maintained significantly and the activities of the antioxidant enzymes were also enhanced by red LED treatment (Jiang et al., 2019). Besides, several studies focused on the postharvest quality of fruits and vegetables stored under red and blue light were carried out (Liu et al., 2025; Ma et al., 2014; Nassarawa & Luo, 2022). It was worth to find that favorable qualities of these food were achieved by using red and blue light. Hence, it could be speculated that red and blue light could also extend the shelf life of fresh-cut sugarcane efficiently. However, information on the influences of red and blue light on storage quality of fresh-cut sugarcane was still limited.

Recently, attentions have been paid to the generation of reactive oxygen species (ROS) including singlet oxygen (^1^O_2_), hydroxyl radical (OH^−^) and hydrogen peroxide (H_2_O_2_) because of its dual effects on food quality. On one hand, ROS formed through photodynamic reaction could result in the damage of protein and DNA damage, the peroxidation of membrane lipid and the disruption of cellular structure of bacterial and fungal to extend the shelf life of food (Ghate, Zhou and Yuk, 2019). On the other hand, excessive accumulation of ROS could also inflict similar damage on the cells of fruits and vegetables and accelerate senescence (Zhou et al., 2020). H_2_O_2_ was deemed to the most stable type of ROS and contributed a lot to the senescence of fruit (Jiang et al., 2019). Recently, the exogenous H_2_O_2_ treatment has been widely applied to food and exhibited remarkable effects on food quality and antioxidative system (Chumyam et al., 2019; Linares-Castañeda et al., 2025). However, there were few reports on the roles of internally generated H_2_O_2_ that induced by light in the physiological, microbial and metabolomics changes after harvested. Moreover, the specific contributions of ^1^O_2_ and OH^−^ to the damages of both microorganisms and food were still limited. Understanding these mechanisms is crucial for the optimization of preservation techniques and quality control of fruit and vegetable.

Due to the strongest oxidability, ^1^O_2_ was also reported to play a critical role on photodynamic sterilization (Feuerstein et al., 2005). As the energy of LED light is transferred directly to oxygen molecules to form ^1^O_2_, reducing the level of O_2_ might be a method to prevent the over accumulation of ROS. Based on the fundamental knowledge and findings in previous studies, it was hypothesized that red and blue light combined with vacuum treatment is able to inhibit microbial growth and nutrients loss in fresh-cut sugarcane. However, the levels of different ROS produced during storage periods, as well as their influences on the quality of fresh-cut sugarcane throughout the storage duration remained largely unclear.

Thus, the aim of this study was to investigate the impacts of red and blue light on the storage quality and metabolomics profiles of fresh-cut sugarcane and to explore the relationships between ROS and these quality indexes. This study offers new insights into the mechanisms behind the quality changes of fresh-cut sugarcane during storage by elucidating the roles of different ROS. The results of this study could provide a theoretical foundation and practical guidance for the application of light irradiation technology for the preservation of fresh-cut sugarcane, as well as other fruits and vegetables.

## 2 Materials and methods

### 2.1 Chemicals and reagents

Sodium hydrate, sodium dihydrogen phosphate, Folin-phenol, trichloroacetic acid (TCA), 2-thiobarbituric acid (TBA), kalium chloratum, rutin, gallic acid, nitityl nitrogen blue tetrazolium (NBT), diphenzo (a, h) anthracene, carbon tetrachloride (DPA), phenol, catechol, p-aminobenzene sulfonic acid, α-naphthylamine and hydroxylamine hydrochloride were analytical grade and purchased from Macklin Biochemical Co., Ltd. (Shanghai, China). 2,2-Diphenyl-1-picrylhydrazyl radicals (DPPH) and 2, 2′-azino-bis (3-ethylbenzthiozoline-6)-sulphonic acid (ABTS) were analytical grade and purchased from Aladdin Biochemical Technology Co. LTD (Shanghai, China). Plate count agar (PCA) and rose bengal medium were purchased from Hope Bio-Technology Co. Ltd. (Qingdao, China). Methanol, formic acid and acetonitrile were of HPLC grade and bought from Thermo Fisher Scientific Co., Ltd. (Shanghai, China).

### 2.2 Sugarcane materials and treatments

The mature green chewing sugarcane without damages on surface were collected from Datian County, Fujian Province, China. Fresh sugarcanes were cut into 15–20 cm immediately after harvest. These fresh-cut sugarcanes were divided into 4 groups, 2 of which were vacuum-packed and the remaining 2 groups were wrapped at each incision using plastic wrap. Thereafter, the packed sugarcane was subjected to a mixture of red-blue light and dark condition for storage, respectively. Thus, these 4 groups were named as PWD (plastic wrapped sugarcane in dark), PWL (plastic wrapped sugarcane in light), VPD (vacuum packed sugarcane in dark) and VPL (vacuum packed sugarcane in light) for short. The light parameters were set as follow: peak wavelength of red light: 615 nm, peak wavelength of blue light: 455 nm, irradiance: 5.6 w/m^2^ and photon flux density: 8.683 umol/(m^2^·s). The vertical distance between sugarcane and light source was 50 cm. Fresh-cut sugarcanes were continuously illuminated for 15 d at 5 °C and turned over at 24 h intervals to ensure uniform irradiation of the light source. Sugarcane samples from each storage condition were collected every 5 d to evaluate the storage quality. Juice of each sample were obtained using an electric sugarcane juicer (YF-T80, Xiuling Kitchen Ware Co., Ltd., Guangdong, China) for further analysis.

### 2.3 Physiological quality assessment

#### 2.3.1 Respiration rate

A fruit and vegetable respirometer (3051H, Top Yunnong Technology Co., Ltd.，Zhejiang，China) was used to determine respiration rate. Results were calculated as the following equation.

$$\mathrm{REP}=\frac{\left(C_{t}-C_{o}\right)\times V\times 1.98\times 10^{-3}}{m\times t}$$

where REP is the respiration rate (mg/ (kg·h)), *C*_*t*_ is the concentration (μL/L) of CO_2_ after test, *C*_*o*_ is the initial concentration (μL/L) of CO_2_, *V* is the volume of testing room, *m* is the weight of sample (g), *t* is the testing time (h), 1.98 × 10^−3^ is the density of CO_2_ (mg/μL).

#### 2.3.2 Weight loss

Weight loss was evaluated by regularly weighing samples at various storage time. The result was calculated according to the following equation,

$$\text{Weight loss}=\left[\left(m_{0}-m_{1}\right)/m_{0}\right]\times 100\%$$

where *m*_*0*_ is the initial weight (g) and *m*_*1*_ is the weight (g) at the specified time point.

#### 2.3.3 Soluble solid content and pH

Soluble solids content (SSC) and pH were defined using extracted juice directly with a hand-held refractometer (PAL-1, ATAGO Co., Ltd., Tokyo, Japan) and a digital pH meter (S20, Mettler Toledo Instrument Co., Ltd., Zurich, Switzerland), respectively.

#### 2.3.4 Color difference

A handheld colorimeter (YS3010, San'enshi Technology Co., Ltd., Shenzhen, China) was used to determine the color parameters (L*, a* and b*) of sugarcane incision. The formula of color difference (∆E) was as follow:

$$∆E=\sqrt{\left[{\left(∆L^{*}\right)}^{2}+{\left(∆a^{*}\right)}^{2}+{\left(∆b^{*}\right)}^{2}\right]}$$

### 2.4 Microbial analysis

Total bacterial counts (TBC) and total yeast and mold counts (YMC) in fresh-cut sugarcane were measured following the Chinese national standard methods (GB 4789.2-2016; GB 4789.2-2022). The microbial counts were expressed as lg CFU/g.

### 2.5 Antioxidant activity assay

Antioxidant activity assays were both performed as the protocol reported by Zhang et al. (2014) with a simple modification. For DPPH scavenging rate determination, 100 μL sugarcane juice was added to 100 μL DPPH free radical solution (0.1 mM methanolic solution of DPPH) and left to stand for 30 min at 25 °C in dark. The absorbance (Abs) of the reaction system was measured at 517 nm using the microplate reader (Synergy H1, Agilent Technology Co., Ltd., Shanghai, China). The scavenging rate of DPPH radicals was calculated by the following equation,

$$\text{DPPH scavenging rate}\left(\%\right)=\left[1-\left(A_{1}-A_{2}\right)/A_{3}\right]\times 100\%$$

where *A*_*1*_ was the Abs of 100 μL DPPH free radical solution with 100 μL sugarcane juice, *A*_*2*_ was the Abs of ethanol with 100 μL sugarcane juice, *A*_*3*_ was the Abs of 100 μL DPPH free radical solution with 100 μL ethanol.

In terms of ABTS scavenging rate, potassium persulfate solution was mixed with ABTS solution (1,1, *v*/v) for 12 h at 5 °C in dark to obtain ABTS stock solution. The deionized water was used to adjust the Abs of stock solution to 0.7 ± 0.02 at 734 nm to obtain the ABTS free radical working solution. Then, 50 μL of sugarcane juice was added to 150 μL of ABTS working solution in a 96-well plate. The mixture was stood for 6 min in darkness, and then subjected into the microplate reader. The measurement was conducted at 734 nm. The scavenging rate of ABTS radicals was calculated by the following equation:

$$\text{ABTS scavenging rate}\left(\%\right)=\left[1-\left(B_{1}-B_{2}/B_{3}\right)\right]\times 100\%$$

where *B*_*1*_ was Abs of 150 μL ABTS free radical working solutions +50 μL sugarcane juice, *B*_*2*_ was the Abs of 150 μL ionic water +50 μL sugarcane juice, *B*_*3*_ was the Abs of 150 μL ABTS free radical solutions +50 μL deionized water.

### 2.6 Analysis of enzyme activities

Extractions and detections of superoxide dismutase activity (SOD), catalase activity (CAT), ascorbate peroxidase activity (APX), peroxidase activity (POD) and polyphenol oxidase activity (PPO) were conducted following the previously reported procedures (Zhang et al., 2015). Firstly, centrifuged sugarcane juice was added to the phosphate buffer saline required for different enzymes, shaken thoroughly and centrifuged again at 4 °C for 15 min. The supernatant was collected for the enzyme activity determination by microplate reader.

The ability of SOD to inhibit the photo reduction of NBT was recorded as its activity. The increase in Abs (560 nm) of 0.01 per minute was used as 1 activity unit (U). The decomposition of H_2_O_2_ in the presence of CAT at 240 nm was measured to analyze the activity of CAT. One unit (U) of CAT activity was defined as the amount of enzyme that decomposes 1 nmol of H_2_O_2_ per minute. APX activity was defined by measuring the oxidation of ascorbic acid in presence of H_2_O_2_. One unit of APX activity was defined as enzymes amount that resulted in Abs changes of 0.01 at 290 nm per minute. For POD and PPO analysis, the increase in Abs of 0.01 per minute was used as 1 activity unit (U). The detection wavelength was 470 nm and 420 nm for POD and PPO, respectively.

### 2.7 Measurements of ROS production and malondialdehyde content

Superoxide anion (O_2_^−^) generation rates were determined using the method of (Zhang et al., 2015) with simple modifications. Juice of fresh-cut sugarcane was centrifuged (10, 000 rpm, 10 min) firstly. The reaction mixture was consisted of 5 mL supernatant, 5 mL of 50 mmol/L phosphate buffer saline (pH = 7.8) and 1 mL of 1 mmol/L hydroxylamine hydrochloride solution. After incubated at 25 °C for 20 min, the mixture was added with 1 mL of p-aminobenzene sulfonic acid solution and 1 mL of α-naphthylamine solution. The Abs were measured at 530 nm by UV Spectrophotometer (V-1100, SHANGHAI MAPADA INSTRUMENT Co., LTD, Shanghai, China). The O_2_^−^ production rate was expressed as nmol/ (g·min).

The content of H_2_O_2_ was determined according to the method described by Zhou et al. (2020). The sugarcane juice was added to 5 mL of cold acetone solution for centrifugation (6000 rpm, 10 min). The supernatant (1 mL) was added with 0.1 mL TiCl_4_-HCl solution, 0.2 mL ammonia water and stood for 5 min. The precipitate was obtained after centrifugation at 8000 rpm for 15 min and repeatedly rinsing with cold acetone until colorless. Dissolved the precipitate with 3 mL H_2_SO_4_ completely and read the Abs at 412 nm by UV Spectrophotometer. The result was expressed as mg/g.

The determination of ^1^O_2_ was performed based on the method of Diwu and Lown (1992) with slight modifications. As the reaction between DPA and ^1^O_2_ in equal volume could produce the stable compound 9, 10-Diphenylanthrac superoxide, the decrease in Abs of DPA at 375 nm recorded by UV Spectrophotometer could represent the production of ^1^O_2_.

Malondialdehyde (MDA) was determined based on the method of Zhang et al. (2015) with some modifications. Added 0.5 mL of sugarcane juice to 10 mL of 10 % TCA, followed by centrifugation (10, 000 rpm, 10 min) at 5 °C. Mixed 3 mL supernatant with 6 mL of 0.6 % TBA and boiled for 20 min. And then centrifuged again after cooling. Samples were recorded at 532 nm and the result was presented as μmol/g based on fresh weight.

### 2.8 Metabolomics analysis

Fresh samples (stored for 0 d, CK) and treated samples stored for 15 d (PWL15, PWD15, VPL15, and VPD15) were ground after frozen at −40 °C for 48 h using a vacuum freeze-dryer (SCIENTZ-10 N, Ningbo Scientz Biotechnology Co., Ltd., Ningbo, China). Then, 800 mg sample mixed with 5 mL of 60 % methanol aqueous solution was sonicate in an ice bath for 40 min. After centrifuged at 12,000 rpm for 20 min, the supernatant was collected and filtered into a sample vial for mass spectrometry analysis. Metabolomics analysis of these extracts was carried out using a HPLC-MS/MS system (ZenoTOF 7600, AB Sciex Analytical Instrument Trading Co., Ltd., MA, USA) coupled with a Atlantis Premier BEH C18 AX (2.1 × 100 mm, 1.7 μm, Waters Corporation, MA, USA). The column temperature was set as 40 °C. The mobile phase consisted of 0.5 mg/L aqueous solution of formic acid (phase A) and acetonitrile (phase B). The gradient program was set as: 0–10 min, 3 % B; 10–13 min, 90 % B; 13–15 min, 3 % B. The flow rate was 0.3 mL/min and injection volume 2 μL. The mass spectrometer was operated in positive/negative polarity mode with a spray voltage of 5500 V, the capillary temperature of 550 °C, a sheath gas flow rate of 55 psi, aux gas pressure of 55 psi. The MS/MS acquisition mass range is 50–1500 Da. Datasets obtained from MS/MS system were imported into the software of OSI/SMMS (Dalian Institute of Chemical Physics, Chinese Academy of Sciences, Dalian, China) for qualitative analysis, principal component analysis (PCA) and differential analysis. The metabolic pathways related to these differential metabolites were analyzed using the KEGG database (https://www.Kegg.jp).

### 2.9 Statistical analysis

All experiments were performed in triplicate. ANOVA test was conducted using SPSS 13.0 statistical software (SPSS Inc., Chicago, USA) to compare the mean values of each treatment. Cluster and Vann analysis were performed by Hiplot Pro (https://hiplot.com.cn/). Advanced Cor link was performed using the OmicStudio tools at https://www.omicstudio.cn/tool.

## 3 Results and discussion

### 3.1 Physiological quality of fresh-cut sugarcane

#### 3.1.1 Appearance and color difference of the incision

**Fig. 1:**
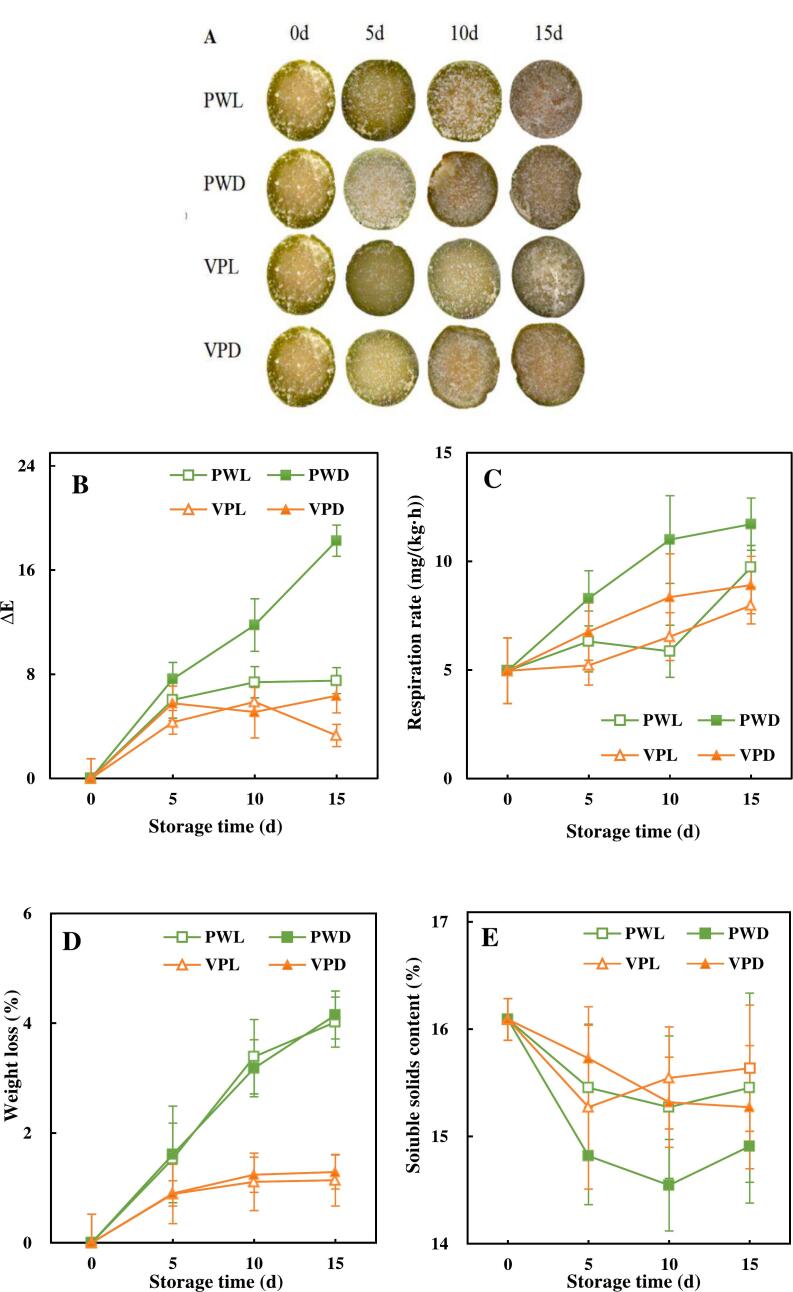

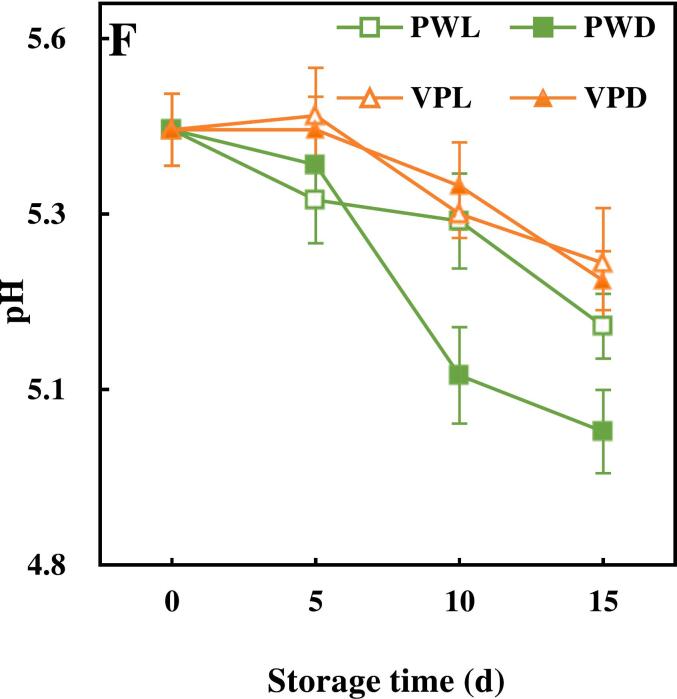
Effects of light conditions and packages on the physiological quality of fresh-cut sugarcane. Graphs in Fig. 1 illustrated the changes of physiological quality of vacuum packed and plastic wrapped fresh-cut sugarcane along with storage time. A. Appearance of fresh-cut sugarcane, B. color difference of the incision, C. Respiration rate, D. Weight loss, E. Soluble solids content, F pH. Points are the means of triplicate samples and error bars represent the standard deviation of the means.

Browning was an obvious phenotype of the quality deterioration of fresh-cut sugarcane. As shown in Fig. 1A, a serious browning on the incision of fresh-cut sugarcane occurred during storage. The sugarcane samples packed with plastic wrap lose the bright yellow appearance and turned to brown since 10 d. Vacuum package could prevent the browning of incision obviously. Seen from the appearance of sugarcane samples (Fig. 1A), both browning and lignification were delayed by vacuum package and light treatment.

In the case of the color change (ΔE) of incision, there was a significant difference between stored sugarcane and fresh sugarcane since 5 d (Fig. 1B). The most pronounced changes in ΔE was observed in PWD, which was increased to 18.25 at the end of storage. The results of ΔE indicated that vacuum packaging combined with light treatment could relieve the color change of fresh-cut sugarcane. These finding were in accord with the results of appearance of fresh-cut sugarcane (Fig. 1A). In addition, the role of red and blue light treatment on color protection in plastic package groups was more obvious than that in vacuum package group. Previous studies have confirmed that light irradiation can enhance the activity of antioxidant enzymes (Yao et al., 2023). Continuous light treatment for 7 d reduced polyphenol oxidase (PPO) activity, which is responsible for enzymatic browning, by approximately 20 % in cauliflower (Yao et al., 2023). Thus, the changes in the activity of enzymes related to browning and anti-browning induced by light treatment might be responsible for the remarkable color- preserving effect observed in this study.

#### 3.1.2 Respiration rate, weight loss, soluble solids content and pH

As shown in Fig. 1C, due to the mechanical damage, the respiration rates (REP) of all fresh-cut sugarcane samples were increased over the storage time except PWL. The REP of vacuum packed sugarcane were slower than that of plastic wrapped, which might result from the low oxygen environment created by vacuum packaging. The apparent reductions of REP were obtained by light treatment. Similarly, LED light also had significant respiratory inhibition effects on pakchoi (Zhou et al., 2020). Thus, red and blue light treatment was an effective method to maintain the nutritional quality of fresh-cut sugarcane.

The weight loss (WL) of fresh-cut sugarcane with vacuum packaging was kept in the range of 1.14 %–1.29 % during the storage period (Fig. 1D). However, the WL of PWL and PWD raised up to 4.02 % and 4.15 % at 15 d, respectively. The results implied that WL of fresh-cut sugarcane could be effectively suppressed by vacuum packaging, and was almost unaltered by the presence or absence of light. Compared to other fresh-cut vegetable and fruits, fresh-cut sugarcane had lower weight loss. As only the incisions of fresh-cut sugarcane were exposed to the air, the low WL might be attributed to the inhibition of transpiration and respiration by the thick rind (Rogiers et al., 2004).

The SSC of fresh-cut sugarcane was dropped first and had no significant difference since 5 d (Fig. 1E). PWD had a significant lower SSC than the other 3 samples. The decreased SSC during early storage might be ascribed to the carbohydrates consumption by wound respiration and microbial spoilage. Light treatment affected SSC significantly (*P* < 0.05). The reduction in SSC consumption of light irradiated samples may be attributed to the inhibition of the accelerated respiration rate by light. These findings revealed that vacuum package and light treatment could regulate SSC and preserve the edible and nutrient quality of fresh-cut sugarcane during the storage. Consistent with our results, red and blue light treatment could also reserve the SSC of peach (Gong et al., 2015).

The initial pH of sugarcane was 5.42. As shown in Fig. 1F, all the samples started to descend from 5 d. The results indicated that vacuum packaging was superior to plastic packaging in maintaining acidity stability of fresh-cut sugarcane. Light treatment had a greater effect on pH of plastic packed samples than that of vacuum packed samples. The decrease in pH may be related to the anaerobic glycolysis as well as the spoilage effect of microorganisms in sugarcane (Singh et al., 2008). The ROS induced by light irradiation have the potential to kill microorganisms to some extent. Therefore, it can be suggested that the decrease of pH in plastic wrapped sugarcane might be delayed due to the reduction in microbial population caused by light irradiation.

### 3.2 Microbial analysis on fresh-cut sugarcane

**Fig. 2:**
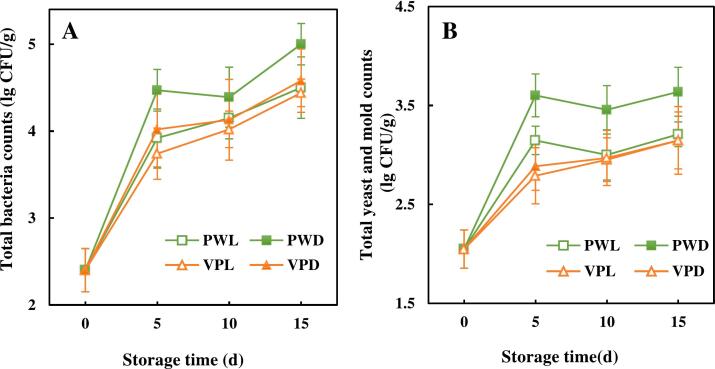
Effects of light conditions and packages on microbial load of fresh-cut sugarcane. Fig. 2 including 2 line charts displayed the changes of total bacteria counts and total yeast and mold counts in vacuum packed and plastic wrapped fresh-cut sugarcane along with storage time. A. Total bacteria counts, B. Total yeast and mold counts. Points are the means of triplicate samples and error bars represent the standard deviation of the means.

It is critical to reduce the number of microorganisms on the surface of fresh-cut sugarcane to ensure its microbiological safety. The change of total bacterial counts (TBC) and total yeast and mold counts (YMC) of fresh-cut sugarcane during storage was illustrated in Fig. 2A and Fig. 2B, respectively. As shown in Fig. 2, TBC and YMC burgeoned in each treatment group during the first 5 days. This result might be attributed to the large amount of juice flowing from fresh-cut sugarcane after mechanical damage, which provided abundant nutrients for microbial growth and reproduction. Both TBC and YMC of PWD were much more than those of the other 3 samples through the whole storage. In addition, there was no significant difference between these 3 samples from the 10th day. At the end of storage, TBC and YMC of PWD increased to 5.00 lg CFU/g and 3.63 lg CFU/g, respectively, while that of the other 3 samples had the TBC in the range of 4.44–4.58 lg CFU/g and the YMC in 3.15–3.21 lg CFU/g (Fig. 2A and B). However, TBC and YMC of all the fresh-cut sugarcane did not exceed the recommended limit of acceptability (6.0 lg CFU/g) during the whole storage period. As indicated by Luo et al. (2015), even though the sugarcane was heated before storage, the TBC and YMC were nearly 4 lg CFU/g by the 5th day and expected to rise up continuously along with the storage time. Whereas, the YMC of sugarcane exposure in light was around 3 lg CFU/g and kept stable to the 15th day in this study. These results suggested that the vacuum package and light irradiation in present study was more efficient in yeast and mold inhibition than thermal treatment. The low TBC and YMC in vacuum packed sugarcane might be due to the inhibition of the growth of aerobic bacteria among the microorganisms by vacuum treatment (Huan et al., 2022). Besides, blue LED illumination combined with natural photosensitizer was found to have an inactivating effect on bacterial pathogens in pineapple slices (Bhavya et al., 2021). Photosensitizer like chlorophyll could be stimulated by light to produce ROS, which could bring about cytotoxic effects by interacting with contiguous intracellular components, such as DNA, proteins and lipids, leading to bacterial death (Ghate et al., 2019). In this study, the chlorophyll existed in green rind of sugarcane might be contributed to ROS formation. Thus, the slow microbial development of sugarcane under light irradiation might be due to the photodynamic reactions that occurred during storage. Furthermore, the antimicrobial effects of light treatment in plastic packed sugarcane was more obvious than those in vacuum packed sugarcane.

### 3.3 Redox status of fresh-cut sugarcane

#### 3.3.1 Antioxidant capacities

**Fig. 3:**
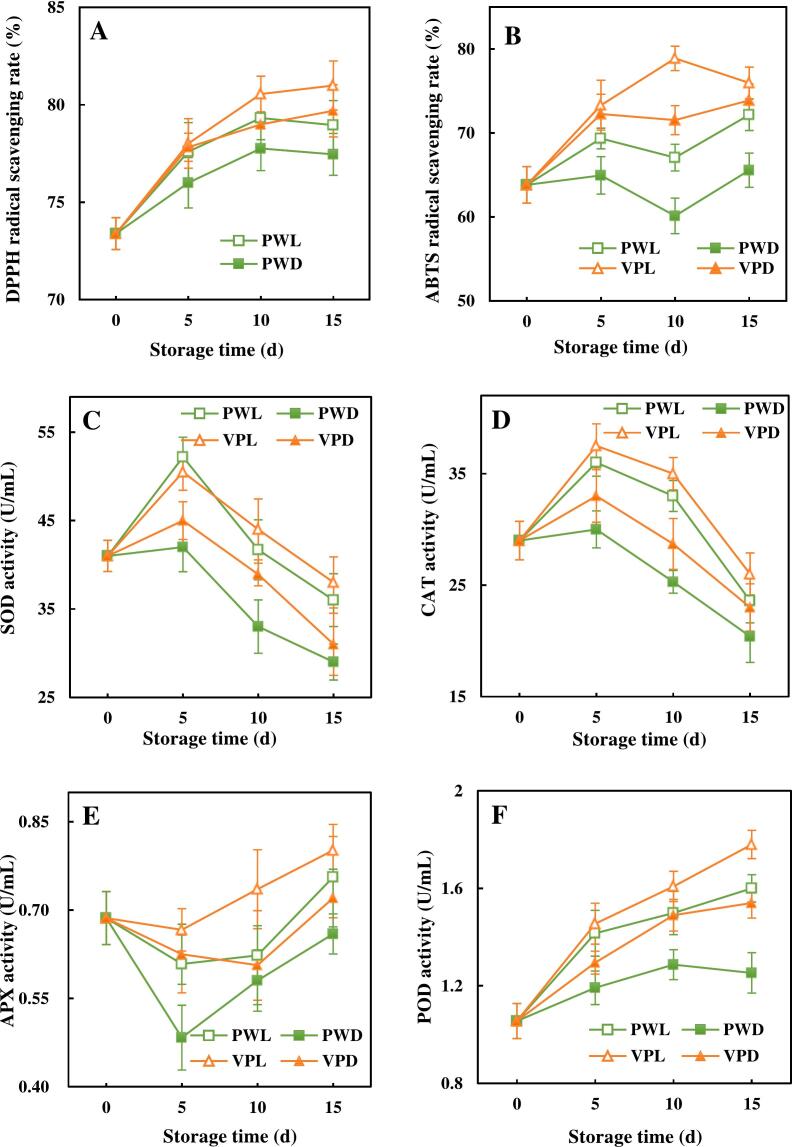

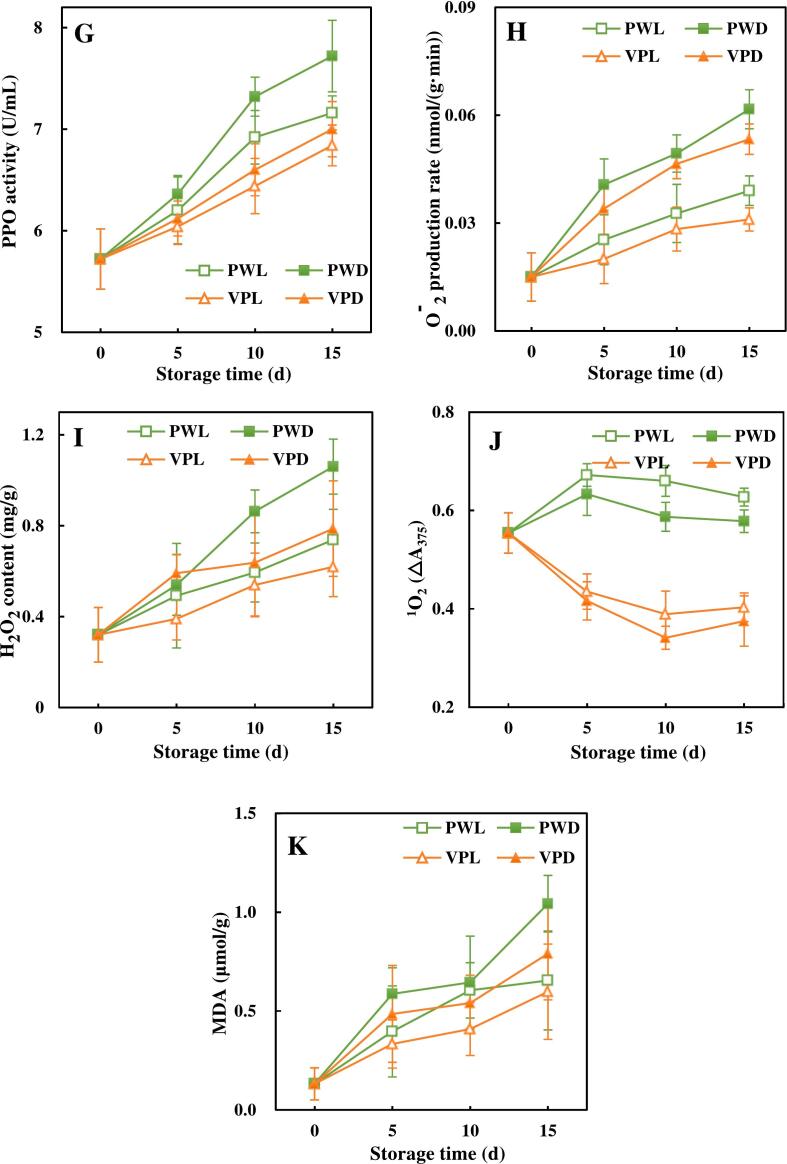
Effects of light conditions and packages on ROS-related characteristics of fresh-cut sugarcane. These charts showed the alterations of antioxidant capacities, enzyme activities, ROS and malondialdehyde in vacuum packed and plastic wrapped fresh-cut sugarcane along with storage time. A. DPPH radical scavenging rate, B. ABTS radical scavenging rate, C superoxide dismutase activity (SOD), D. Catalase activity (CAT), E. Ascorbate peroxidase activity (APX), F. Peroxidase activity (POD), G. Polyphenol oxidase activity (PPO), H·O_2_^−^ production rate, I. H_2_O_2_ content, J. ^1^O_2_ relative production, K. malondialdehyde (MDA) content. Points are the means of triplicate samples and error bars represent the standard deviation of the means.

The impacts of light conditions and packages on the antioxidant capacities of fresh-cut sugarcanes were evaluated by a combination of DPPH and ABTS (Fig. 3A and B). The DPPH of VPL and VPD showed an overall increasing trend, while PWL and PWD increased first and maintained at 81.23 % and 79.02 % after 10 d, respectively. The trend of ABTS in the VPL was increasing in the first 10 d and then started to decrease, while the other 3 groups undulated slightly throughout the storage. It has been reported that the contents of antioxidant substances in fruits and vegetables were positively correlated with their antioxidant capacities (Zhang et al., 2014). Both DPPH and ABTS of PWL and VPL were higher than those of PWD and VPD. This result was probably due to the fact that light stimulated the synthesis of phenolics and flavonoids (Crupi et al., 2013).

#### 3.3.2 Enzyme activities

In order to investigate the antioxidant system of fresh-cut sugarcane with different light conditions and packages, the activities of active oxygen scavenging enzyme including the SOD, CAT, APX, POD and PPO were assayed. As shown in Fig. 3C and D, the activities of SOD and CAT exhibited the similar trends, which increased rapidly and then started to decline from 5 d of storage. Contrary to SOD and CAT, APX activities of fresh-cut sugarcane were firstly decreased and then increased a long with storage time (Fig. 3E), while the variation rates of APX activities in these 4 treatments were quite different. In terms of POD and PPO, consistent growths were observed in fresh-cut sugarcane regardless of treatments (Fig. 3 F and G). Furthermore, exposing sugarcane to red and blue light resulted in higher activities of SOD, CAT, APX and POD than those of sugarcanes stored in darkness. Similar increased in these antioxidant enzyme activities were also found in broccoli and grapes applied with red or blue light irradiation (Jiang et al., 2019; Nassarawa et al., 2022). SOD, CAT and APX had the abilities to scavenge ROS. As a result, activities of these enzyme could be enhanced by ROS induced by light. It was undeniable that vacuum packaging also stimulated the expression of antioxidant enzyme activities and retarded their decline compared to plastic packaging. The antioxidant enzyme activities of fresh-cut peach and mangosteen preserved in vacuum packaging were also higher than that of the air storage groups (Denoya et al., 2015; Manurakchinakorn et al., 2004). On the contrary, light treatment and vacuum package played negative roles on PPO activities of sugarcane during the storage. It has been suggested that the notable suppressed PPO activities in model systems and apple derivatives might be due to the irreversible structural changes of PPO induced by high intensity visible light treatment (Manzocco et al., 2009). This might also be responsible for the reduction of PPO activities observed in sugarcane samples exposed to blue and red light in this study.

#### 3.3.3 Reactive oxygen species and malondialdehyde

As shown in Fig. 3H and I, O_2_^−^ production rate and H_2_O_2_ content were increased all through the storage period. At the end of storage, the levels of these two ROS were decreased in the following order: PWD > VPD > PWL > VPL. For ^1^O_2_, the PW groups initially increased then decreased slightly, while the VP groups decreased rapidly at the beginning and rise again slowly from the 10th day (Fig. 3J). MDA as the peroxidation production of unsaturated fatty acid on the membrane by H_2_O_2_ had the similar trend with H_2_O_2_ naturally (Fig. 3K). Light treatment was beneficial to ^1^O_2_ generation, but had opposite effects on O_2_^−^, H_2_O_2_ and MDA formation. Jiang et al. (2019) and Nassarawa and Luo (2022) also found that the MDA, H_2_O_2_ and O_2_^−^ contents were lower in broccoli and grape treated with red-light than those in dark. The irradiation of red and blue light with the help of endogenous natural photosensitizer in plant could lead to the formation of ROS including O_2_^−^, H_2_O_2_ and ^1^O_2_ (Ghate et al., 2013). The inhibition of O_2_^−^, H_2_O_2_ and MDA in light treated sugarcane could be explained by the enhanced antioxidant systems by the synthesis of phenolic and flavonoids and the activation of antioxidant enzyme (Bhavya et al., 2021). The activated enzymes by light treatment like SOD, CAT, APX and POD could accelerate the conversion of O_2_^−^ and H_2_O_2_ to H_2_O, while almost had no effects on ^1^O_2_. These were reasons why light treatment had different impacts on ^1^O_2_, O_2_^−^ and H_2_O_2_.

### 3.4 Metabolic profiles in fresh-cut sugarcane

**Fig. 4:**
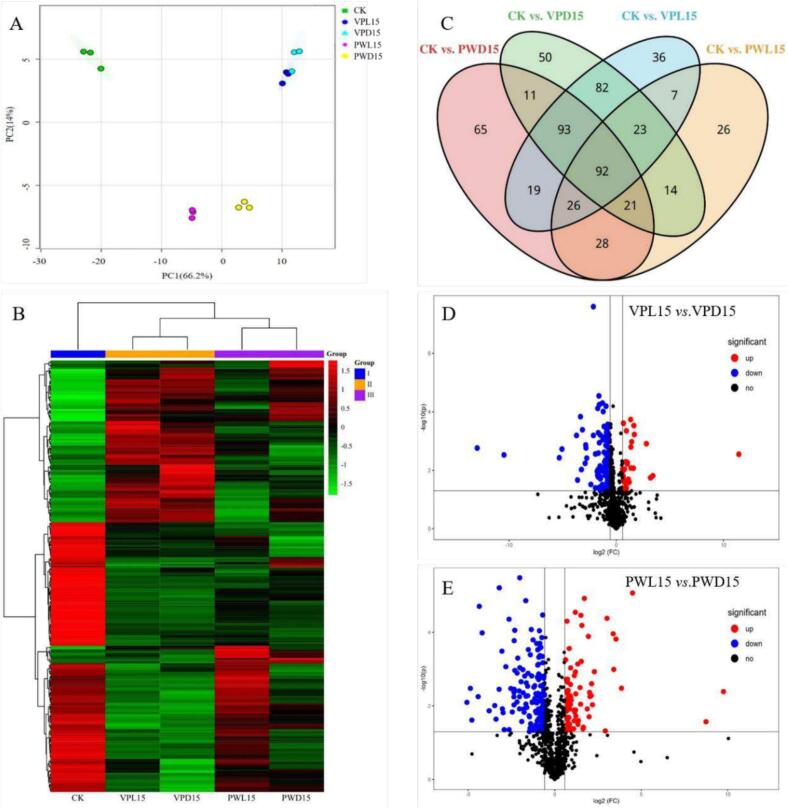
Multivariate statistical analysis of metabolites in control (0 d) and stored sugarcane (15 d). In order to evaluate the differences in the metabolites of fresh-cut sugarcane under different storage conditions, the results of PCA analysis, cluster analysis, Venn analysis and Volcano plots among the control (0 d) and stored sugarcane (15 d) were summarized in Fig. 4. A. Principal component analysis (PCA) score plot, B. Hierarchical cluster analysis of identified differential metabolite in sugarcane, C Overlapping metabolites by Venn analysis among four pairwise comparisons groups. D. Volcano plots between VPL15 and VPD15, E. Volcano plots between PWL15 and PWD 15. CK. fresh sugarcane stored for 0 d.

A total of 805 kinds of known metabolites were identified in this study. In order to evaluate the differences in the metabolites of fresh-cut sugarcane under different storage conditions, a PCA analysis of CK (0d), PWL15, PWD15, VPL15, and VPD15 was conducted (Fig. 4A). CK located far away from the other 4 stored samples in the PCA plots, indicating storage time impacted on the metabolites of fresh-cut sugarcane significantly. Meanwhile, samples with the same package were clustered together, demonstrating that packaging treatments also had great influence a certain impact on the metabolic profile of fresh-cut sugarcane. In consistent with the result of PCA, the 5 samples were divided into 3 groups in the heatmap of hierarchical cluster analysis based on 248 differential metabolites (Fig. 4B). CK was distinguished as the first category, 2 vacuum-packed samples constituted the second category, and 2 plastic-wrapped samples were the third category. In total, 355, 237, 386, and 378 differential metabolites were identified in CK *vs* PWD15, CK *vs* PWD15, CK *vs* VPL15, and CK *vs* VPD15. Seen from the Venn diagram (Fig. 4C), 92 overlapping metabolites were detected in these 4 comparison groups.

In order to gain insights into the effects of light irradiation on metabolic profiles, the comparative changes of metabolites betweenVPL15 and VPD15, as well as PWL15 and PWD15, were illustrated in volcano plots (Fig. 4D and E). In the vacuum packaging group, 70 differential metabolites were obtained including 37 that were upregulated and 33 were downregulated. There were 211diffential metabolites induced by light irradiation in group of PWL15 and PWD15, of which 76 were upregulated and 135 were downregulated significantly. Thus, the influence of red and blue light on the metabolic profiles of plastic wrapped sugarcane was more pronounce than that of vacuum packed sugarcane. This finding was also consistent with the result of PCA.

**Fig. 5:**
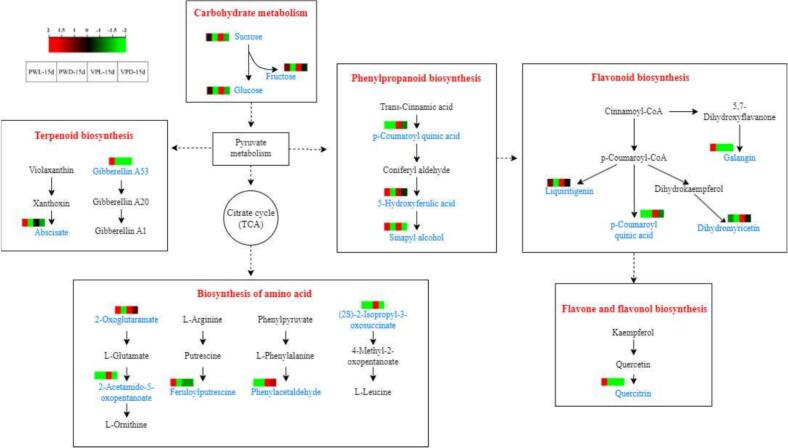
Schematic diagram of the metabolite changes occurred in sugarcane samples of PWD (plastic wrapped in dark), PWL (plastic wrapped in light), VPD (vacuum packed in dark) and VPL (vacuum packed in light) after stored for 15 d. The schematic diagram revealed 5 metabolite pathways associated with the quality of vacuum packed and plastic wrapped fresh-cut sugarcane, integrated with the variation heatmap of the critical metabolites.

After KEGG analysis, 5 metabolic pathways associated with the quality of fresh-cut sugarcane were revealed. The schematic diagram of the metabolite pathways integrated with the variation heatmap of the critical metabolites was presented in Fig. 5. Firstly, the hydrolysis of sucrose into glucose and fructose was a common sugar metabolic pathway in food. It has been confirmed that soluble sugars could reduce the quality deterioration of fruits during storage due to stress from adverse conditions (Yu et al., 2016). The contents of sucrose, fructose and glucose in fresh-cut sugarcane subjected to light treatment were much higher than those without light treatment. As most glucose and fructose were consumed in respiration process, the low respiration rates objected in light treated sugarcane could explore the accumulation of sugars. In terms of molecular mechanism, the expression of some genes related to carbohydrate metabolism might also lead to the changes in the composition and content of sugars. For example, transcripts of three sucrose phosphate synthase (MrSPS1, MrSPS2, and MrSPS3) in Chinese bayberry fruit were markedly induced by blue light treatment throughout the storage period (Shi et al., 2016).

Secondly, metabolite products of fresh-cut sugarcane related to amino acid metabolism exhibited obvious variation after storage. As one of the precursor substances for ornithine synthesis, 2-oxoglutaramate showed an upward trend in sugarcane fruits under light irradiation. However, 2-acetamido-5-oxopentanoate and (2*S*)-2-isopropyl-3-oxosuccinate, the intermediates of amino acids metabolisms, were only upregulated in VPL15 d and downregulated in the other 3 samples. The conversion of feruloylputrescine from arginine was also activated by red and blue light irradiation. Phenylacetaldehyde, as a metabolite product of phenylalanine, was significantly increased in vacuum packaged samples, while decreased in plastic wrapped samples. These amino acids and related bioactive compounds were significantly enriched under red and blue light irradiation, which usually contributes to the maintenance of cell membrane stability, regulating the structure of nucleic acids, modulating the activities of enzymes and scavenging free radicals (Ballester et al., 2006). Thus, the quality of fresh-cut sugarcane might be maintain *via* the accumulation of these functional substances.

A variety of phenolic compounds and lignin are produced in the phenylpropanoid biosynthesis pathway, which play a significant role in regulating plant growth, resisting adverse enviromental stress, and defending against pathogenic invasions (Ballester et al., 2006). p-Coumaroyl quinic acid, 5-hydroxyferulic acid and sinapyl alcohol involved in the biosynthesis of phenylpropanoids were detected in sugarcane under different storage conditions. p-Coumaroyl quinic acid is not noly an intermediate in the phenylpropanoid synthetic pathway but also a precursor for the biosynthesis of flavonoids and terpenoid compounds. As shown in Fig. 5, p-coumaroyl quinic acid was only increased in the VPL15d significantly. 5-Hydroxyferulic acid and sinapyl alcohol were accumulated in all sugarcane exposed under red and blue light. As sinapyl alcohol is one of the key monomers for lignin polymerization, its upregulation is beneficial for the deposition of lignin at the cut surfaces of sugarcane. As a result, weight loss, browning degree and the risk of microbial infection could be reduced. The reduced ΔE and browning degree observed in sugarcane subjected to red and blue light (Fig. 1) could be explained in molecular metabolic perspective.

In the metabolic network of flavonoids, 5 differential metabolites under various storage conditions including liquiritigenin, p-coumaroyl quinic acid, dihydromyricetin, galangin, and quercitrin were identified in fresh-cut sugarcane. Liquiritigenin and dihydromyricetin exhibited a decrease in PWD15, whereas an increase was observed in the other samples. Galangin and quercitrin showed a significant upregulation in PWL15. p-Coumaroyl quinic acid was downregulated in plastic wrapped group but upregulated in the vacuum packaging group. These findings suggested that light exposure and vacuum packaging conditions were more conducive to the biosynthesis of flavonoids to defense against the light stress and oxidative stress. It has been reported that the flavonoid-deficient varieties of crops are more sensitive to UV stress (Ormrod et al., 1995; Stapleton & Walbot, 1994). As reported in previous study (Miao et al., 2016), the expression levels of 12 structural genes involved in flavonoid biosynthesis in strawberries varied significantly under red, blue, yellow, green and white light conditions. However, the preference analysis of transcriptome in fresh-cut sugarcane under red and blue light treatment remained limited.

There were significant differences in the levels of abscisic acid (ABA) and gibberellin A53 (GA53), which are involved in terpenoid biosynthesis, across various sugarcane samples. The upregulation of ABA was induced by light treatment effectively. GA53 was only detected in PWL15. As reported in previous studies, the activation of ABA metabolic gene could promote the formation of callus tissue around the wounded areas of fruits and vegetables, thereby inhibiting the rot caused by pathogen infection (Wang et al., 2024; Wei et al., 2024). Whether red and blue light can upregulate the expression of genes related to ABA signaling in fresh-cut sugarcane remains to be demonstrated. Therefore, the increase in ABA content in light treated sugarcane might be another reason for their low microbial count and high quality.

### 3.5 Roles of ROS on extending the shelf life of fresh-cut sugarcane

**Fig. 6:**
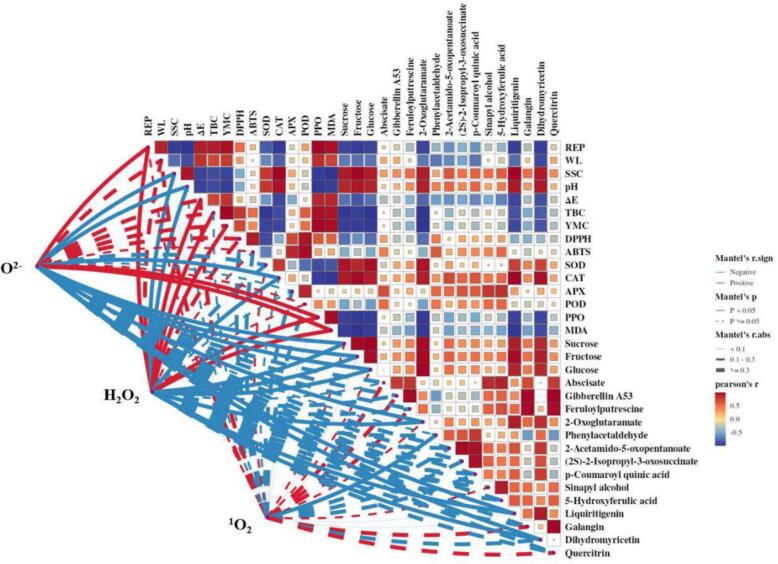
Correlations between ROS and 32 indicators involved in physiological quality, microbial analysis, redox status and differential metabolites of fresh-cut sugarcane. This graph illustrated the relationships between ROS and physiological quality, microbial count, redox status and differential metabolites of fresh-cut sugarcane. Pairwise comparisons of indicators are shown with a color gradient denoting the Pearson's correlation coefficient. Red square represents positive correlation and blue square is negative correlation. The deeper red or blue represented higher correlation values. *P < 0.05, **P < 0.01. The roles of O_2_^−^, H_2_O_2_, and ^1^O_2_ was compared with the each indicators by Mantel tests. REP, respiration rate; WL, weight loss; SSC, soluble solids content; ΔE, color difference; TBC, total bacterial counts; YMC, total yeast and mold counts. SOD, superoxide dismutase activity; CAT, catalase activity; APX, ascorbate peroxidase activity; POD, peroxidase activity; PPO, polyphenol oxidase activity.

ROS had strong oxidizing effects causing the damage of cellular macromolecules like proteins, lipids and nucleic acids, and finally resulting in bacterial death and senescence of plants simultaneously (Ghate et al., 2019). To further examine the roles of ROS in the changes of physiological quality, microbial count, redox status and differential metabolites, a correlation analysis was conducted in this study (Fig. 6). For physiological quality of fresh-cut sugarcane, O^2−^ might lead to the increase of REP and the decrease of SSC and pH. H_2_O_2_ played similar roles with O^2−^ in physiological quality, while also exerted additional effects on ΔE.

The visible correlation results also demonstrated that the influence of O^2−^ and H_2_O_2_ on the indicators of redox status and differential metabolites were highly consistent. Both O^2−^ and H_2_O_2_ were negatively correlated with CAT activity, sucrose, fructose, glucose, 2-oxoglutaramate, liquiritigenin and dihydromyricetin, whereas positively with PPO activity and MDA. These results indicated that these two ROS might be primarily responsible to the oxidative damages of fresh-cut sugarcane. The positive effects of H_2_O_2_ on PPO activity and MDA and negative effects on CAT activity were also found in longan fruit samples during storage (Lin et al., 2020, 2021). Furthermore, CAT and PPO might actively respond to ROS stress. Sugars could provide energy for physiological activities of both microorganisms and sugarcane. 2-Oxoglutaramate is one of the critical precursors of ornithine, and may play a role in enhancing post-harvest stress resistance (Anwar et al., 2018). Liquiritigenin is a major precursor for some flavonoid compounds such as flavanols, and anthocyanins (Akram et al., 2021; Hu et al., 2020). Dihydromyricetin has a variety of biological activities, including anti-inflammatory and antioxidant (Liu et al., 2019). Presumably, the consumptions of the 3 metabolites might contribute to defending the oxidative stress caused by O^2−^ and H_2_O_2_. It was also notable that H_2_O_2_ was significantly positively correlated with TBC and YMC. In fact, the light irradiation and vacuum package could not kill the microorganisms in this investigation. ^1^O_2_ was thought to be an active ROS with strong oxidative capacity and selectivity (Min & Boff, 2002). However, no significant correlation was observed between ^1^O_2_ and the indicators of physiological quality, microbial count as well as redox status. The only metabolites exhibited a negative correlation with ^1^O_2_ was phenylacetaldehyde, which was involved in metabolite pathways of amino acids. That might be attributed to the selective quenching of ^1^O_2_ by some nature compounds (Kong et al., 2021), although the presences of these nature compounds in sugarcane remains to be determined. Thus, biosynthesis of sugars, amino acids and flavonoids were the principal metabolite pathways that corresponding to oxidative stress in fresh-cut sugarcane during storage. It has been confirmed that the alterations of sugar metabolism, amino acid metabolism and flavonoid metabolism induced in harvested fruit and vegetable by light irradiation were related to ROS accumulation (Lee et al., 2020; P. Wang, Chen, et al., 2020; Yan et al., 2022). However, due to the disparities of food types and experimental designs, the changes of detected metabolites in previous literatures were quite different with those in sugarcane. Moreover, studies on the correlations between ROS and these metabolites were still limited. Based on the results of correlation analysis in this study, it could be concluded that the concentration of ROS, especially O^2−^ and H_2_O_2_, should be appropriate to kill bacteria and retain the quality of fresh-cut sugarcane.

## 4 Conclusion

The current study revealed that the combination of red and blue light irradiation and vacuum packaging was effective in extending the shelf life of fresh-cut sugarcane during storage. Firstly, the increases of browning, color change and respiration rate and the decreases of pH in fresh-cut sugarcane were delayed by vacuum package and light treatment obviously. Secondly, TBC and YMC of PWD increased to 5.00 lg CFU/g and 3.63 lg CFU/g, respectively, while that of the other 3 samples had the TBC in the range of 4.44–4.58 lg CFU/g and the YMC in 3.15–3.21 lg CFU/g at the end of the storage. Both DPPH and ABTS of PWL and VPL were higher than those of PWD and VPD. In case of enzyme activities, SOD, CAT and APX, POD were enhanced by light. Light treatment was beneficial to ^1^O_2_ generation, but had opposite effects on O_2_^−^, H_2_O_2_ and MDA formation. There were 211diffential metabolites induced by light irradiation in group of PWL15 and PWD15 and 70 differential metabolites in VPL15 and VPD15. Five metabolic pathways involving sugar, amino acids, phenylpropanoids, flavonoids and terpenoids were indentified. Both O^2−^ and H_2_O_2_ were negatively correlated with CAT activity, sucrose, fructose, glucose, 2-oxoglutaramate, liquiritigenin and dihydromyricetin, whereas positively with PPO activity and MDA. ^1^O_2_ was highlighted because of its notability negative correlation with phenylacetaldehyde. The biosynthesis of amino acids and flavonoids were the principal metabolite pathways that corresponding to oxidative stress in fresh-cut sugarcane during storage. It could be concluded that the concentration of ROS, especially O^2−^ and H_2_O_2_, should be appropriate to kill bacteria and retain the quality of fresh-cut sugarcane. The results of this study were useful for understanding the roles of light irradiation and ROS during food storage. Nonetheless, the best levels of ROS for extending the shelf life of fresh-cut sugarcane and analysis of the transcriptome still need further investigation.

## Funding

This work was supported by 10.13039/501100005270Fujian Provincial Department of Science and Technology (2023J01480), the earmarked fund for 10.13039/501100012453CARS (grant no. CARS-17), National Engineering Research Center of Sugarcane (NERD202231), Nanping City Department of Science and Technology (N2023T029), Special Project for the Central-guided Local Science and Technology Development (2022L3086).

## CRediT authorship contribution statement

**Lu Wang:** Writing – review & editing, Writing – original draft, Project administration, Funding acquisition. **Zhengrong Lin:** Investigation, Formal analysis. **Cheng Peng:** Formal analysis. **Hua Zhang:** Funding acquisition. **Lulu Zhang:** Investigation. **Shoujing Zheng:** Writing – review & editing, Supervision. **Jiebo Chen:** Supervision, Project administration.

## Declaration of competing interest

The authors declare that they have no known competing financial interests or personal relationships that could have appeared to influence the work reported in this paper.

## Data Availability

Data will be made available on request.
